# Supplementary material for: Structural basis for activation of DNMT1
Source: Nat Commun. 2022 Nov 21;13:7130. doi: 10.1038/s41467-022-34779-4 (PMC9681727; doi:10.1038/s41467-022-34779-4)
Supplement: Supplementary file 1 — Supplementary Information [file 41467_2022_34779_MOESM1_ESM.pdf]

Supplementary Information

for

Structural basis for activation of DNMT1

Amika Kikuchi, Hiroki Onoda, Kosuke Yamaguchi, Satomi Kori, Shun Matsuzawa, Yoshie Chiba, Shota Tanimoto, Sae Yoshimi, Hiroki Sato, Atsushi Yamagata, Mikako Shirouzu, Naruhiko Adachi, Jafar Sharif, Haruhiko Koseki, Atsuya Nishiyama, Makoto Nakanishi, Pierre-Antoine Defossez, Kyohei Arita

email: aritak@yokohama-cu.ac.jp

Supplementary Notes

Supplementary Note 1. Structural analysis of DNMT1 bound to double monoubiquitinated H3

To examine the structural rearrangements of the DNMT1 protein upon binding of ubiquitinated H3 during enzymatic activation, we used small angle X-ray scattering (SAXS) and cryo-EM single particle analysis.

Histone H3 monoubiquitinated at K18 and K23 was prepared by linking the G76C ubiquitin by disulfide bond with H3 N-terminal tail harboring K18C/K23C (hereafter H3Ub2$^{s\text{-}s}$) [1]. We performed *in vitro* DNA methylation assay and found that binding of H3Ub2$^{s\text{-}s}$ significantly enhanced the activity of DNMT1 (aa: 351-1616) (Supplementary Fig. 1e). Next, we conducted size exclusion chromatography in line with SAXS (SEC-SAXS) of apo-DNMT1 and DNMT1 bound to H3Ub2$^{s\text{-}s}$ (Supplementary Fig. 6b, 8a-c, and Supplementary Table 3). The radius of gyration (R_g) of apo-DNMT1 as estimated from Guinier analysis was 39.0 Å, and the scattering curve was well superimposed on the curve calculated from the crystal structure of human apo-DNMT1 (PDB: 4WXX). This indicated that in solution, apo-DNMT1 formed an autoinhibitory structure (Supplementary Fig. 6b left). The R_g of the DNMT1 bound to H3-Ub2$^{s\text{-}s}$ was modestly larger (43.7 Å) than that of the apo-DNMT1. However, the scattering curve of DNMT1 bound to H3Ub2$^{s\text{-}s}$ was nearly identical to that of a model structure of DNMT1:H3Ub2 complex obtained by manual docking of RFTS:H3Ub2 structure (PDB: 5WVO) on the corresponding moiety of the crystal structure of apo-DNMT1. These results indicated that double monoubiquitinated H3 binding does not lead to displacement of the RFTS domain from the catalytic domain (Supplementary Fig. 6b right).

To further validate the above model, we performed cryo-EM single particle analysis of apo-DNMT1 bound to ubiquitinated H3. We first attempted preparation of the binary complex using DNMT1 and H3Ub2$^{S\text{-}S}$; however, preparation of the cryogenic sample-grid suitable for atomic-resolution analysis failed under oxidative conditions. To overcome this problem, we performed *in vitro* ubiquitination and prepared isopeptide-linked ubiquitinated H3. H3 tail (residues 1-37W) containing the mutations K14R/K27R/K36R, prepared in house, was ubiquitinated using E1, E2 and UHRF1 (hereafter H3Ub2, see method). As expected, the binary complex DNMT1:H3Ub2 purified by a gel-filtration chromatography showed ubiquitinated H3-dependent DNA methylation activity (Supplementary Fig. 1d). Furthermore, 3.4 Å resolution cryo-EM map of apo-DNMT1 showed structural similarity with the crystal structure of apo-DNMT1 except for the flexible states of the CXXC domain and N-lobe of the RFTS domain (Supplementary Fig. 6a left). C-lobe of RFTS domain was embedded into the catalytic core, indicating an autoinhibitory state (Supplementary Fig. 6a left) [2,3]. 3.6 Å resolution of cryo-EM map of the DNMT1 in complex with H3Ub2 also revealed that the binding of H3Ub2 increased the dynamics of N-lobe of RFTS domain, resulting in complete invisibility of N-lobe bound to H3Ub2 (Supplementary Fig. 6a right). The density of N-lobe of the RFTS domain was obscured in the presence and absence of the H3Ub2 due to conformational flexibility of a long-α helix connecting the two lobes [4]. However, the C-lobe was still accommodated in the catalytic core of the DNMT1:H3Ub2 complex.

Collectively, these data indicate that the RFTS domain is not completely sequestered from the catalytic domain when H3Ub2 binds to the RFTS domain. However, the structural perturbation between

the RFTS and catalytic domains in DNMT1 upon binding of H3Ub2 was enough for the hemimethylated DNA to penetrate into the active center of the catalytic domain.

Supplementary Note 2. Recognition DNA$^{mCG/fCG}$ by DNMT1 in the ternary complex

DNA$^{mCG/fCG}$ duplex is embedded into the catalytic core of the DNMT1, and that the 5-fluorocytosine is flipped-out from the DNA duplex (Supplementary Fig. 5b). In addition, the reactive Cys1226 forms a covalent bond with the 6-carbon of 5-fluorocytosine, while the 5-carbon was found to be protruding out from the π-plane of the pyrimidine ring of 5-fluorocytosine (Supplementary Fig. 5b). The cryo-EM map also captures the conjugation step of 5-fluoro substituent by Cys1226, immediately after transfer of the methyl substituent from SAM (Supplementary Fig. 5b). The resultant S-adenosyl-homocysteine (SAH) was observed at 3.4 Å, away from transferred methyl substituent. The catalytic loop containing residues 1224-1238, including the reactive Cys1226, is inserted into the DNA minor groove at the mCG/fCG site. Arg1234 recognizes the 2-carbonyl group of 5-methlcytosine, while the CH-π cluster residues (Arg1234-Phe1235-Arg1238) pushes out the 5-methlcytosine from the major groove (Supplementary Fig. 5c, d). The exposed methyl group of 5-methylcytosine in DNA$^{mCG/fCG}$ duplex is surrounded by the hydrophobic residues, Cys1499, Leu1500, Trp1510, Leu1513 and Met1533 of the TRD (Supplementary Fig. 5c). Recognition of mCG/fCG sequence by the catalytic loop and the TRD is similar to that in the previously reported DNMT1:DNA$^{mCG/fCG}$ structures [5–7] .

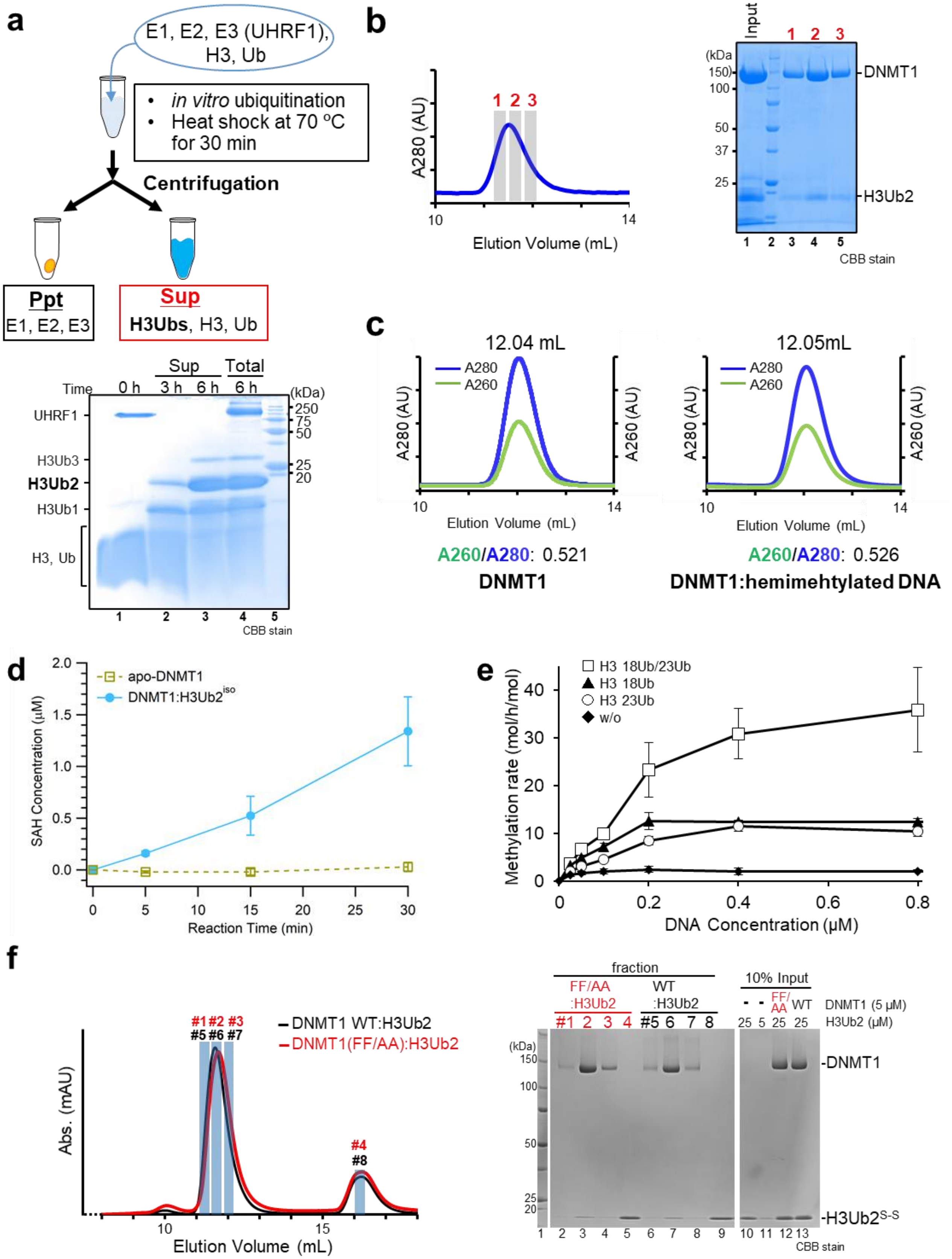
a
E1, E2, E3 (UHRF1), H3, Ub
in vitro ubiquitination
Heat shock at 70 °C for 30 min
Centrifugation
Ppt
E1, E2, E3
Sup
H3Ubs, H3, Ub
Time
0 h
Sup
3 h
6 h
Total
6 h
(kDa)
250
75
50
25
20
UHRF1
H3Ub3
H3Ub2
H3Ub1
H3, Ub
1
2
3
4
5
CBB stain
b
A280 (AU)
1 2 3
10
14
Elution Volume (mL)
Input
1 2 3
(kDa
150
100
50
37
25
DNMT1
H3Ub2
1 2 3 4 5
CBB stain
c
12.04 mL
A280
A260
A280 (AU)
A260 (AU)
10
14
Elution Volume (mL)
A260/A280: 0.521
DNMT1
12.05mL
A280
A260
A280 (AU)
A260 (AU)
10
14
Elution Volume (mL)
A260/A280: 0.526
DNMT1:hemimehtylated DNA
d
apo-DNMT1
DNMT1:H3Ub2iso
SAH Concentration (μM)
2.0
1.5
1.0
0.5
0.0
0
5
10
15
20
25
30
Reaction Time (min)
e
H3 18Ub/23Ub
H3 18Ub
H3 23Ub
w/o
Methylation rate (mol/h/mol)
40
30
20
10
0
0
0.2
0.4
0.6
0.8
DNA Concentration (μM)
f
#1 #2 #3
#5 #6 #7
DNMT1 WT:H3Ub2
DNMT1(FF/AA):H3Ub2
#4
#8
Abs. (mAU)
10
15
Elution Volume (mL)
fraction
FF/AA :H3Ub2
WT :H3Ub2
#1 2 3 4
#5 6 7 8
10% Input
- - FF/AA WT
DNMT1 (5 μM)
25 5 25 25
H3Ub2 (μM)
(kDa)
150
100
50
25
20
DNMT1
H3Ub2S-S
1 2 3 4 5 6 7 8 9 10 11 12 13
CBB stain

Supplementary Figure 1. Sample preparation and biochemical assay.
(a) Schematic figure of *in vitro* preparation of ubiquitinated H3. Lower panel shows analysis of ubiquitination of histone $H3_{1\text{-}37W}$ by SDS-PAGE. Lane 1: ATP omitted-reaction cocktail, lanes 2 and 3: Supernatant (sup) of *in vitro* ubiquitination reaction cocktail for 3 h and 6 h after heat-shock at 70 ºC, lanes 4: Total proteins in the reaction cocktail for 6 h reaction before the heat shock, lane 5: size marker. Source data are provided as a Source Data file. **(b)** Size exclusion chromatography of the mixture of $DNMT1_{351\text{-}1616}$ and excess ubiquitinated H3. Vertical and horizontal axes indicate the absorbance of 280 nm (blue line: A280) and elution volume of Superdex® 200 Increase 10/300 GL, respectively. SDS-PAGE of input (lane 1) and elution fractions were depicted in the chart of size exclusion chromatography. Source data are provided as a Source Data file. **(c)** Size exclusion chromatography of apo-DNMT1 (left) and apo-DNMT1 mixed with hemimethylated DNA ($DNA^{mCG/fCG}$) after conjugating reaction for 12 hr at 25 ºC (right). A260/A280 value at the peak is shown, indicating that DNMT1 was unable to form the binary complex with the DNA. Source data are provided as a Source Data file. **(d)** DNA methylation assay of DNMT1 (aa:351-1616) in the absence of and presence of $H3Ub2^{iso}$. Vertical and horizontal axes indicate SAH concentration and reaction time, respectively. Line shows the average value of three times independent experiments. Data are presented as mean values ± SD for n = 3. Source data are provided as a Source Data file. **(e)** DNA methylation assay of apo-DNMT1 (black-square) and DNMT1 in the presence of H3K18Ub (black-triangle), H3K23Ub (white-circle) and H3K18Ub/K23Ub (white-square). At least three independent experiments were performed for estimation of standard deviation (error bars represent ±SD of n=3). Source data are provided as a Source Data file. **(f)** Left: Size exclusion chromatography of the mixture of wild type (WT) or mutant (FF/AA: F631A/F632A) $DNMT1_{351\text{-}1616}$ in the presence of excess $H3Ub2^{S\text{-}S}$. Vertical and horizontal axes indicate the absorbances at 280 nm and elution volume from Superdex® 200 Increase 10/300 GL, respectively. Right: Analysis of complex formation of WT or FF/AA mutant DNMT1 with $H3Ub2^{S\text{-}S}$ by SDS-PAGE. Lanes 10-13 show the input sample for the SEC analysis. Source data are provided as a Source Data file.

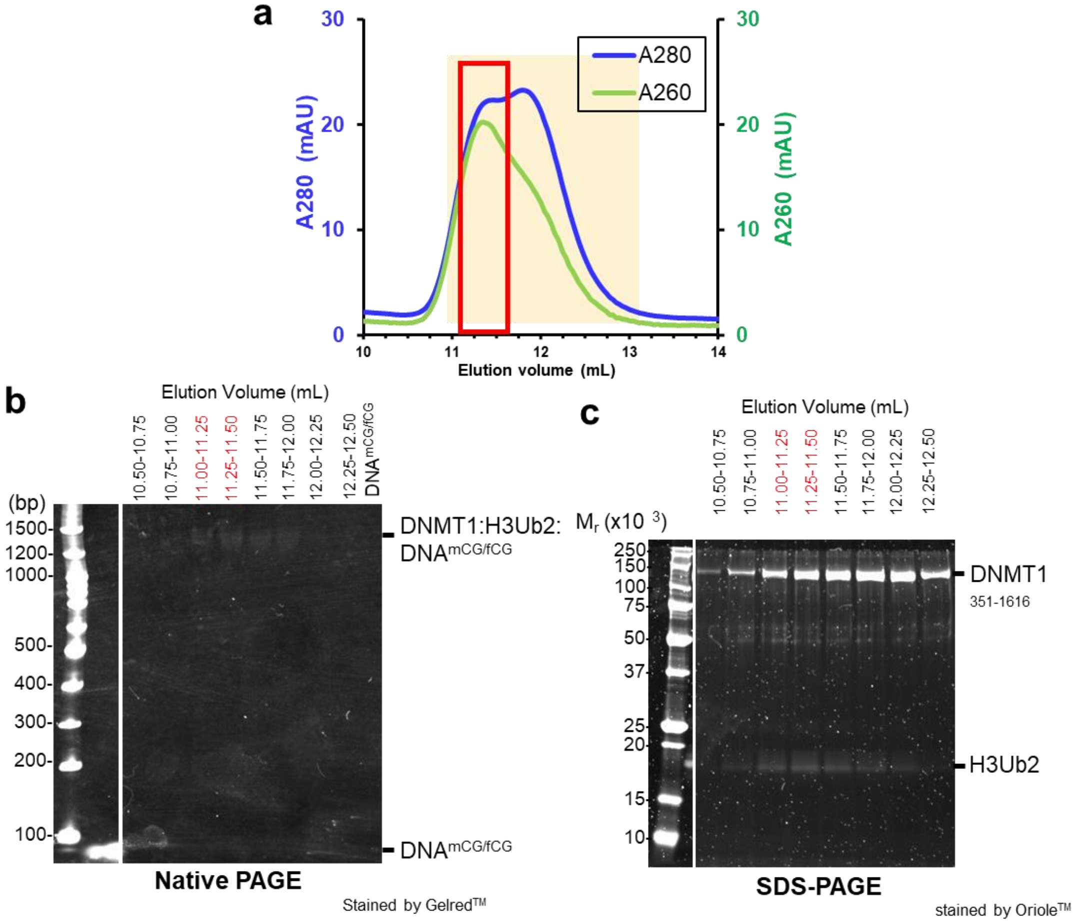

Supplementary Figure 2. Preparation of DNMT1:H3Ub2:DNA$^{mCG/fCG}$ for cryo-EM.

(**a**) Size exclusion chromatography of the mixture of DNMT1:H3Ub2 binary complex and DNA$^{mCG/fCG}$. The area highlighted in yellow was analyzed by electrophoresis, and red box indicates the ternary complex used for cryo-EM analysis. Source data are provided as a Source Data file. All fractions in the chromatography peaks were analyzed by **(b)** Native-PAGE stained by Gelred^{TM} for detecting DNA and **(c)** SDS-PAGE stained by Oriole^{TM} for detecting proteins. Source data are provided as a Source Data file.

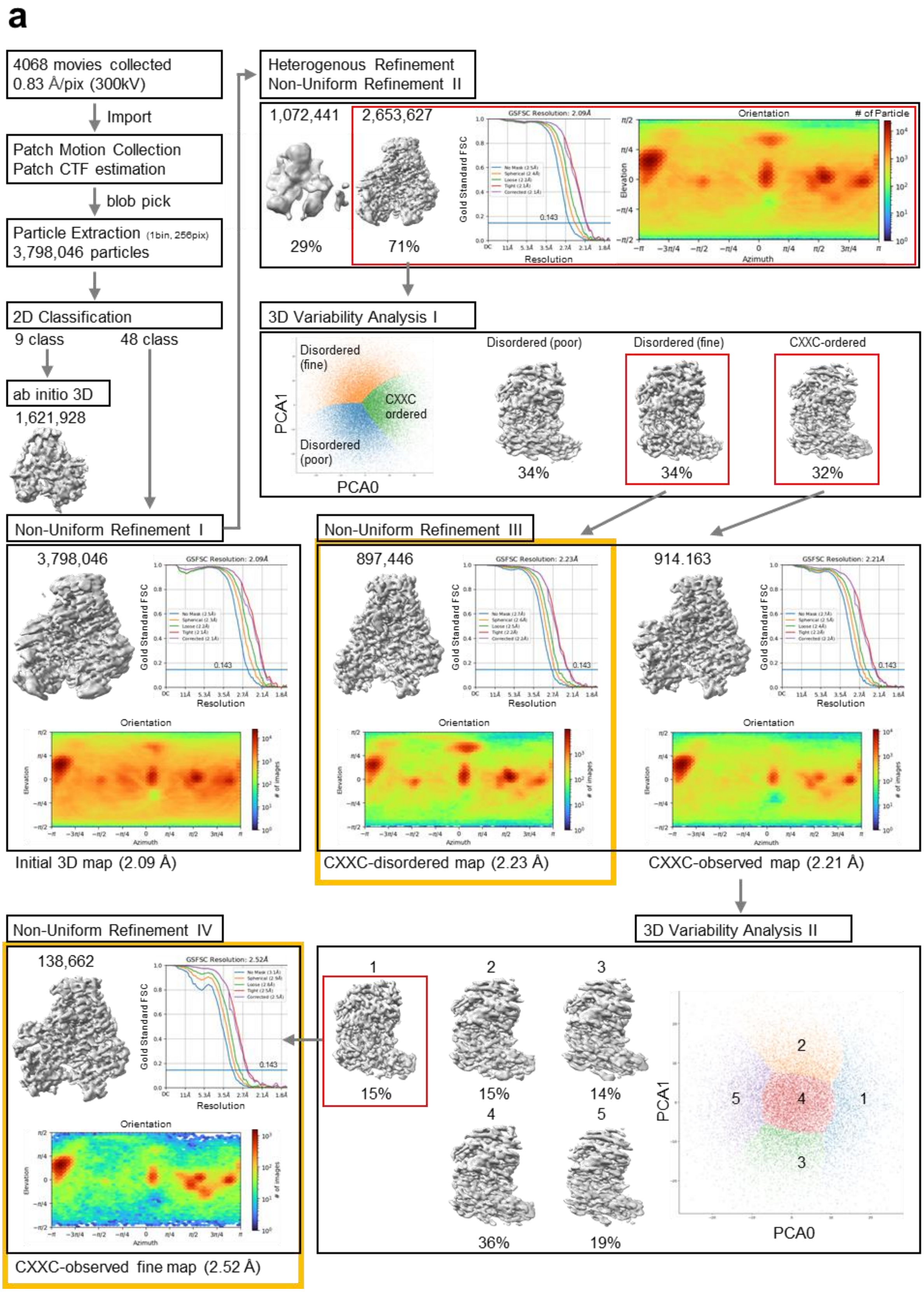

a
4068 movies collected
0.83 Å/pix (300kV)
Import
Patch Motion Collection
Patch CTF estimation
blob pick
Particle Extraction (1bin, 256pix)
3,798,046 particles
2D Classification
9 class
48 class
ab initio 3D
1,621,928
Non-Uniform Refinement I
3,798,046
Initial 3D map (2.09 Å)
Heterogenous Refinement
Non-Uniform Refinement II
1,072,441
2,653,627
29%
71%
3D Variability Analysis I
Disordered (fine)
CXXC ordered
Disordered (poor)
PCA1
PCA0
Disordered (poor)
Disordered (fine)
CXXC-ordered
34%
34%
32%
Non-Uniform Refinement III
897,446
CXXC-disordered map (2.23 Å)
914.163
CXXC-observed map (2.21 Å)
3D Variability Analysis II
1
2
3
15%
15%
14%
4
5
36%
19%
Non-Uniform Refinement IV
138,662
CXXC-observed fine map (2.52 Å)
Resolution
Orientation
Azimuth
Elevation
Gold Standard FSC
0.143

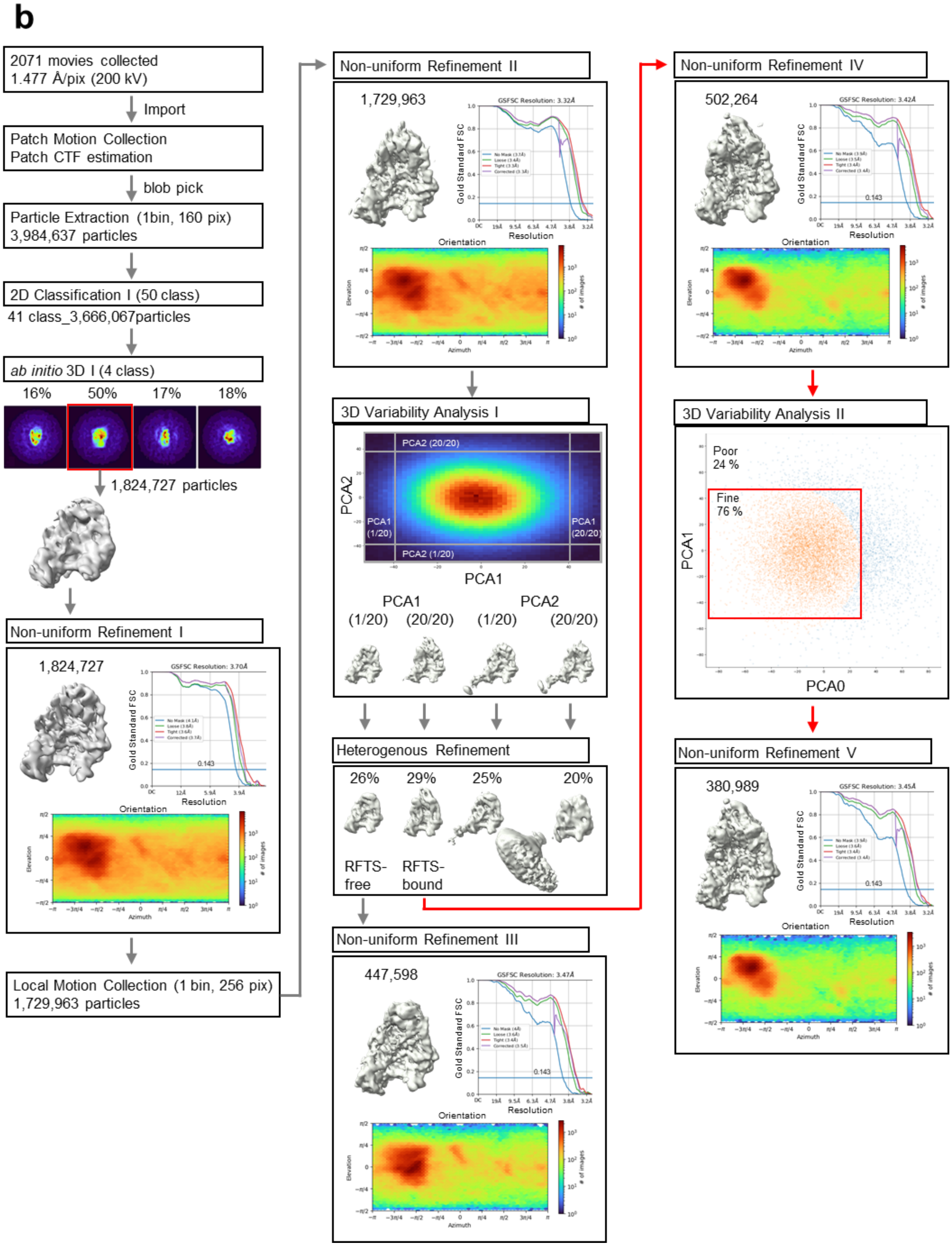
b
2071 movies collected
1.477 Å/pix (200 kV)
Import
Patch Motion Collection
Patch CTF estimation
blob pick
Particle Extraction (1bin, 160 pix)
3,984,637 particles
2D Classification I (50 class)
41 class_3,666,067particles
ab initio 3D I (4 class)
16%
50%
17%
18%
1,824,727 particles
Non-uniform Refinement I
1,824,727
Non-uniform Refinement II
1,729,963
Non-uniform Refinement IV
502,264
3D Variability Analysis I
PCA2
PCA1
PCA1 (1/20) (20/20)
PCA2 (1/20) (20/20)
3D Variability Analysis II
Poor
24 %
Fine
76 %
PCA0
Heterogenous Refinement
26%
29%
25%
20%
RFTS-free
RFTS-bound
Non-uniform Refinement V
380,989
Non-uniform Refinement III
447,598
Local Motion Collection (1 bin, 256 pix)
1,729,963 particles
Resolution
Orientation
Gold Standard FSC
Elevation
Azimuth
0.143

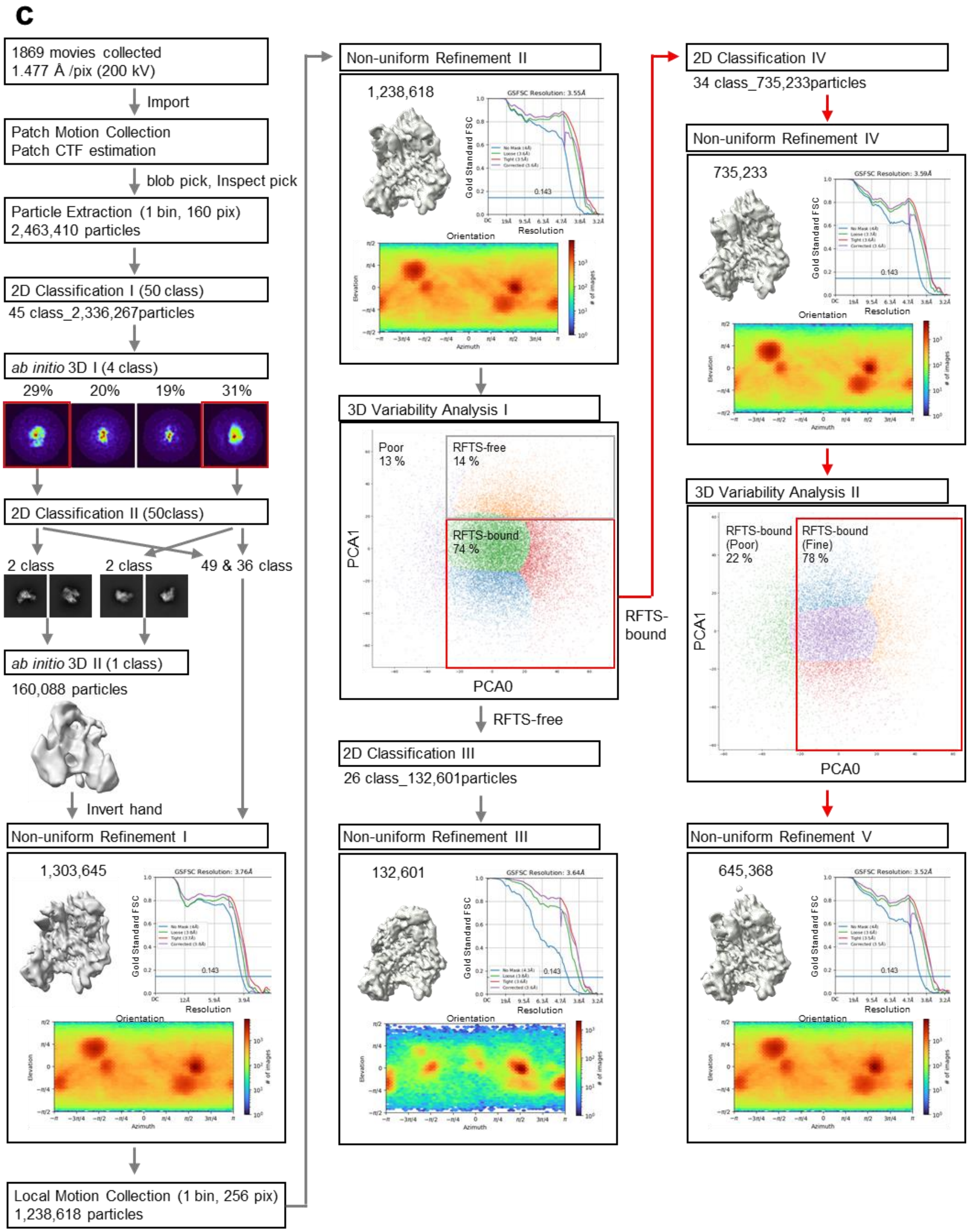

Supplementary Figure 3. Overview of cryo-EM data processing and refinement workflow of (a) DNMT1:H3Ub2:DNA$^{mCG/fCG}$, (b) DNMT1:H3Ub2 and (c) apo-DNMT1. The resolution was estimated based on the Fourier shell correlation (FSC) curve according to the 0.143 criterion.

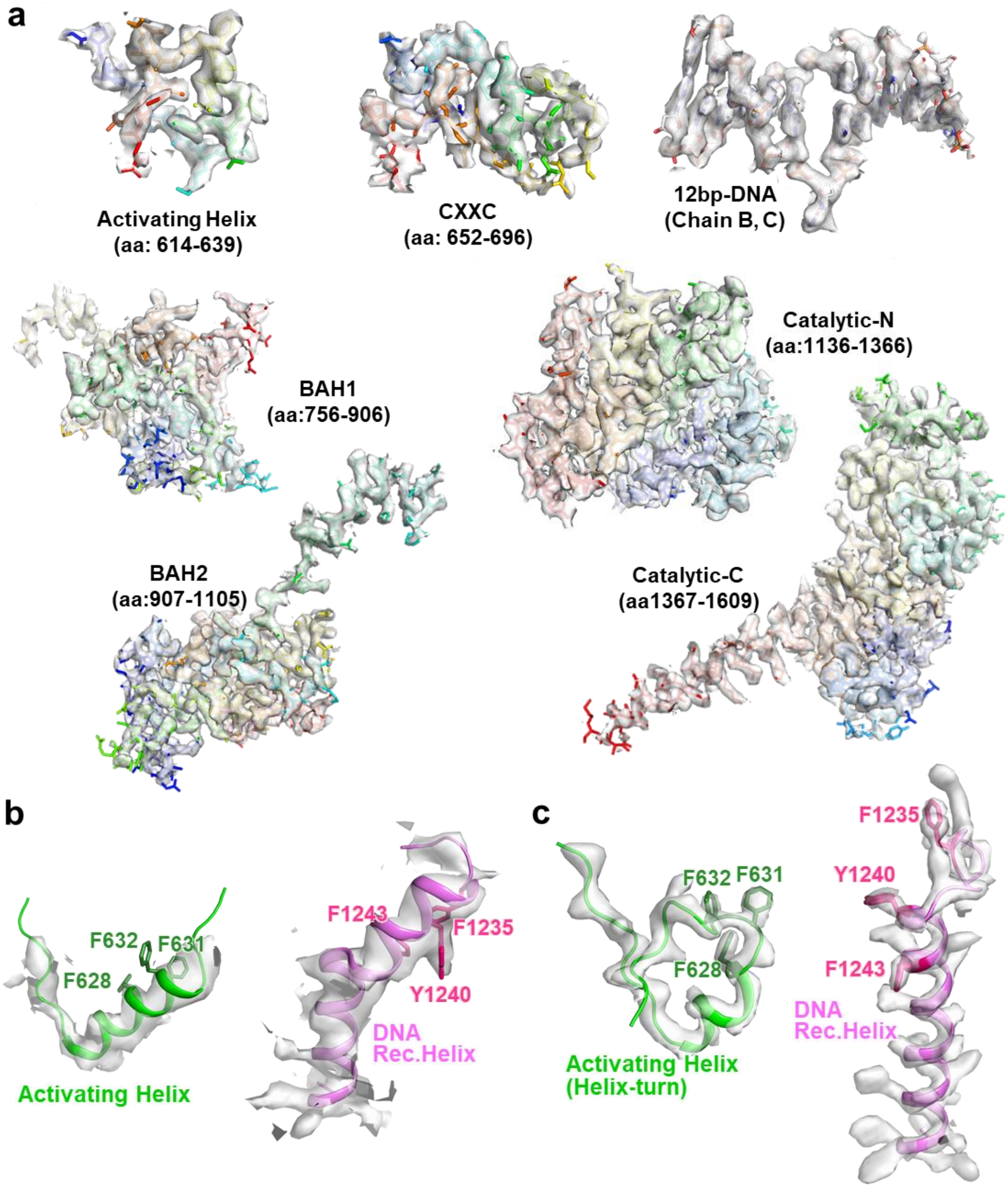

Supplementary Figure 4. Cryo-EM maps of CXXC-ordered DNMT1:H3Ub2:DNA$^{mCG/fCG}$.

(a) The atomic models of DNMT1 and DNA are superimposed on the cryo-EM map. **(b)** Cryo-EM map of Activating Helix and DNA Recognition Helix of apo-DNMT1 superimposed on crystal structure of apo-DNMT1 (PDB:4WXX) **(c)** Structure of Activating Helix and DNA Recognition Helix in the ternary complex superposed on cryo-EM map.

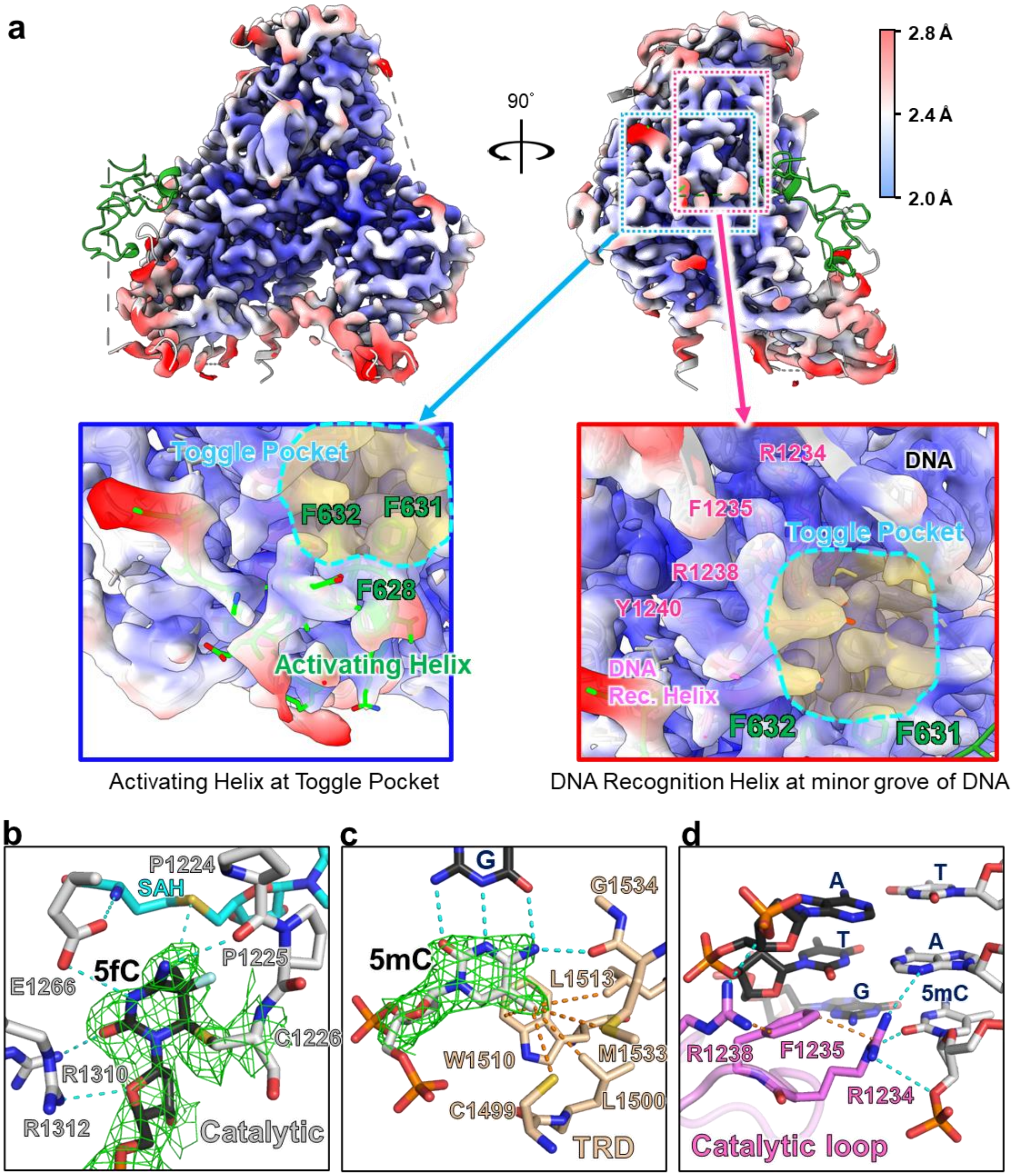

Supplementary Figure 5. Local resolution map of CXXC-disordered DNMNT1:H3Ub2:DNA$^{mCG/fCG}$.

(a) CXXC-disordered cryo-EM map of transparent surface were overlayed on the cartoon model of CXXC-ordered model by UCSF Chimera. Cryo-EM map from front view (upper left) and side view (upper right) were colored with the local resolution, which is estimated by cryoSPARC. Close-up view of the Toggle Pocket (lower left) and minor groove of DNA at mCG/fCG site (lower right) were depicted in lower panel. Stick model of CXXC-ordered model were shown in same color scheme with Fig. 1b. **(b)** Recognition of 5-fluolocytosine (5fC). 5fC, amino acid residues in the catalytic domain of

DNMT1 and SAH are shown as black, gray and cyan stick models, respectively. Cyan dotted line indicates hydrogen bond. Green mesh shows cryo-EM map. **(c)** 5-methylcytosine (5mC) recognition by TRD domain of DNMT1 showing light orange stick model. Cryo-EM map corresponding to 5mC was shown as green mech. **(d)** Recognition of the DNA from the minor groove at 5mCG/5fCG site by catalytic loop of DNMT1. The residues in the catalytic loop are shown as pink stick model.

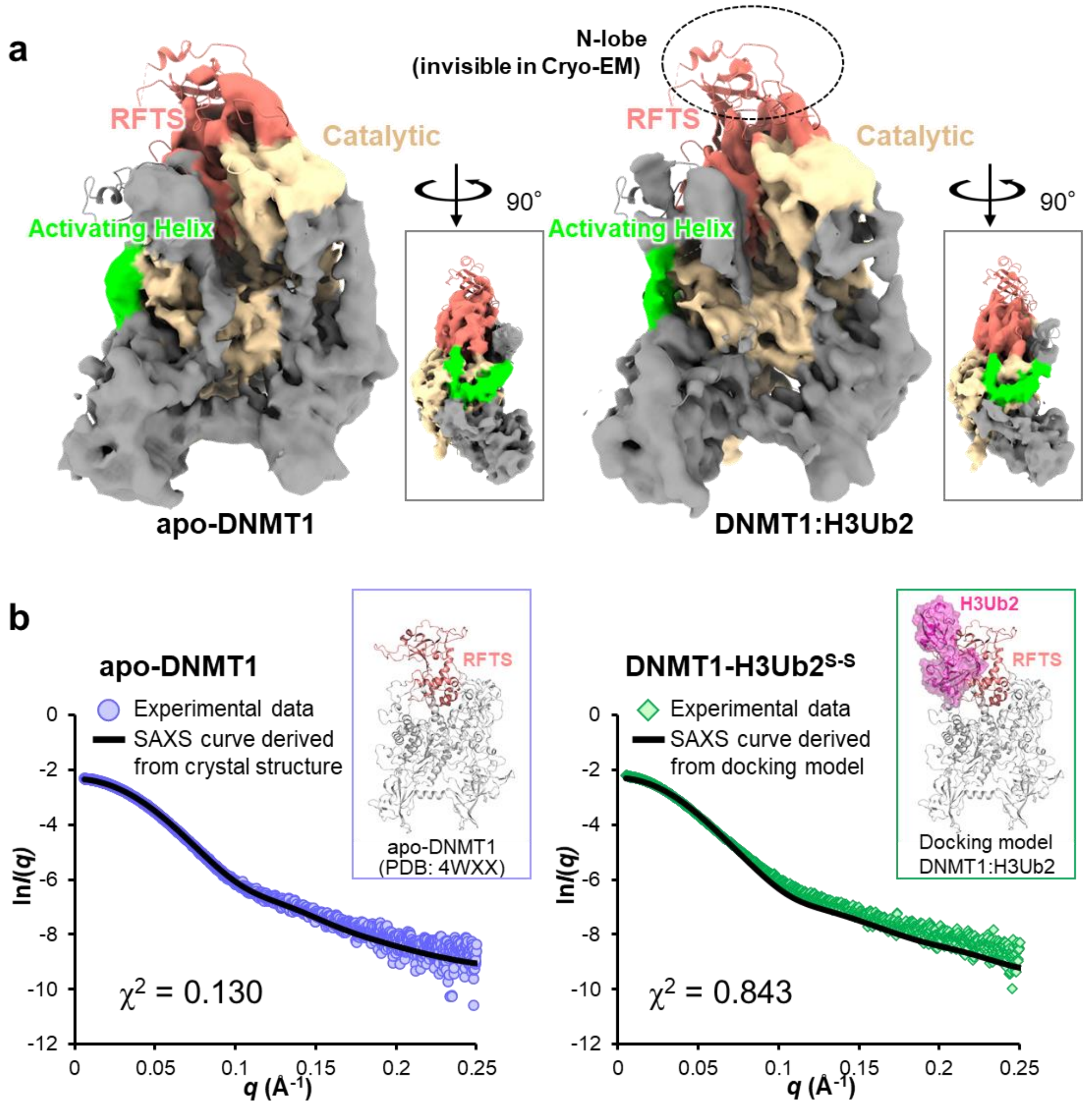

Supplementary Figure 6. Structural analysis of apo-DNMT1 and its in complex with H3Ub2.

(a) Cryo-EM map of apo-DNMT1 (aa:351-1616, left) and the DNMT1:H3Ub2 binary complex (right). RFTS, Activating Helix and catalytic domains are colored salmon pink, green and beige, respectively. The cartoon model of crystal structure of apo-DNMT1 (PDB: 4WXX) is superimposed on the cryo-EM maps. **(b)** SAXS intensity data of apo-DNMT1 (purple circle) and DNMT1:H3Ub2^{S-S} complex (green square) superimposed on the theoretical scattering curves, showing black line, derived from crystal structure of apo-DNMT1 (PDB: 4WXX) and a model structure of apo-DNMT1 docking with RFTS:H3Ub2 complex (PDB:5WVO), respectively. The inset depicts the cartoon model of apo-DNMT1 and the docking model of DNMT1:H3Ub2 complex. Source data are provided as a Source Data file.

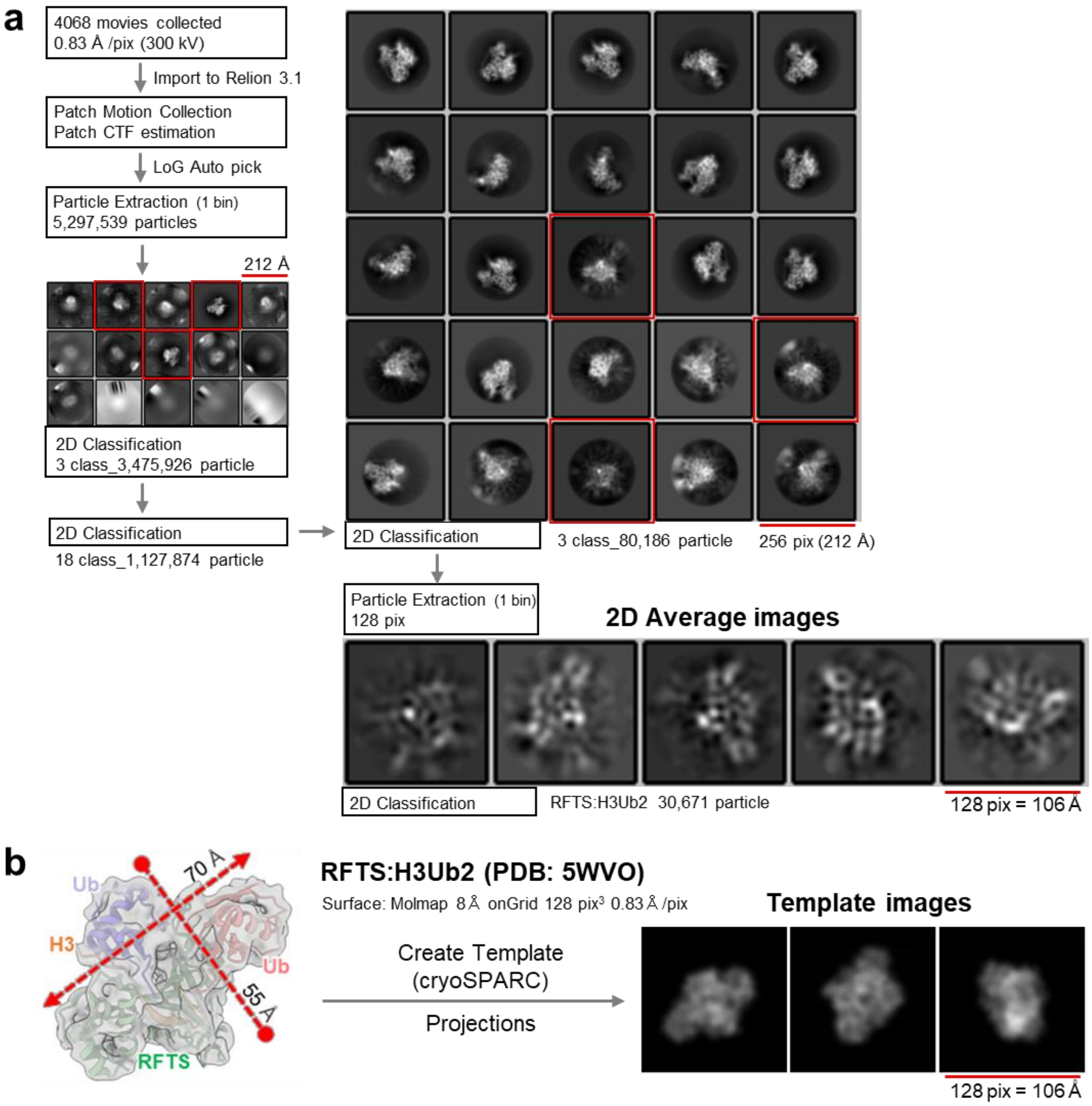

Supplementary Figure 7. Cryo-EM analysis of DNMT1:H3Ub2:DNA$^{mCG/fCG}$ using RELION-3.1. **(a)** Data processing were performed on RELION-3.1. 3,624,260 particles were automatically picked by crYOLO from 4,068 motion corrected movies. After the reference-free 2D classification, *ab initio* 3D model was constructed from the best nine in the 2D classes. The hand flipped *ab initio* 3D model of apo-DNMT1 were used for the initial model. DNMT1 map was obtained after the single round of 2D classification and 2 rounds of Refine 3D. The final resolution was estimated as 2.3 Å by postprocess of RELION with soft mask. Single particle image was also extracted by LoG Auto picker of RELION to check the particles of other biomolecules. After three rounds of 2D classification, 80,186 particles smaller than DNMT1 were selected and re-extracted in a box size of 128 pixels with a 0.83 Å/pixel size, and the images were classified by 2D classification. **(b)** The major 2D average images were compared with the projected templates of H3Ub2-RFTS complex (PDB: 5WVO). Gaussian model of the complex was created by the Molmap of ChimeraX. The 2D projected templates were created by the module of “create template” in cryoSPARC.

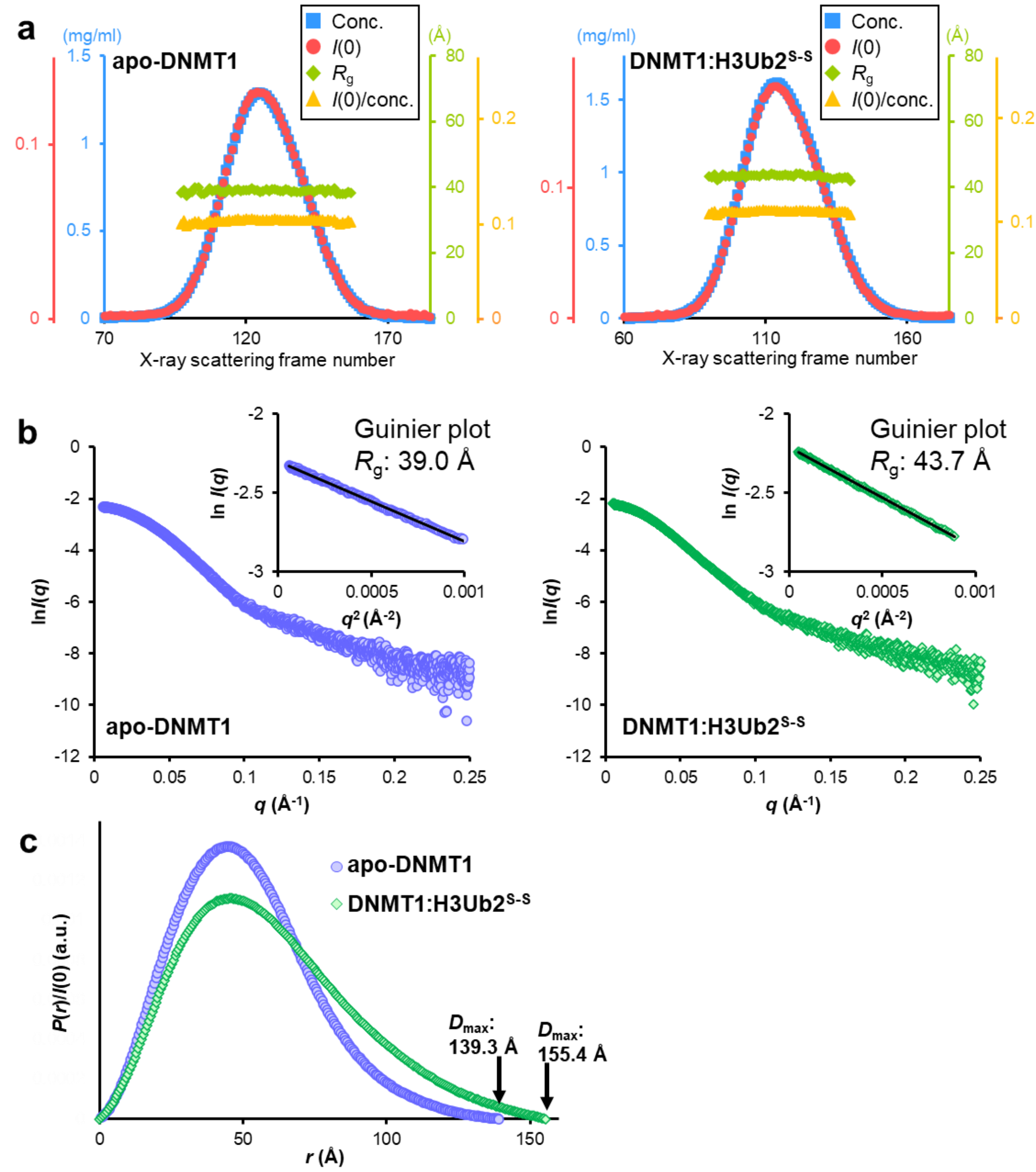

Supplementary Figure 8. SAXS data analysis.
(a) Sample concentration (blue), $I(0)$ (red), R_g (green) and $I(0)/c$ (yellow) plots for SEC-SAXS of apo-DNMT1 (left) and DNMT1:H3Ub2$^{S\text{-}S}$ (right). **(b)** Experimental X-ray scattering curves of apo-DNMT1 (left) and DNMT1:H3Ub2$^{S\text{-}S}$ (right). Vertical and horizontal axes indicate absolute intensity $\ln I(q)$ and scattering angle $q = 4\pi\sin\theta/\lambda$, respectively. Inset indicates Guinier plot with showing R_g value. **(c)** Pair distance distribution functions $P(r)$ of apo-DNMT1 (purple) and DNMT1:H3Ub2$^{S\text{-}S}$ (green) determined from SAXS data. The $P(r)$ functions were normalized by $I(0)$ calculated from each scatter plot. Source data of Supplementary Figs 8a-c are provided as a Source Data file.

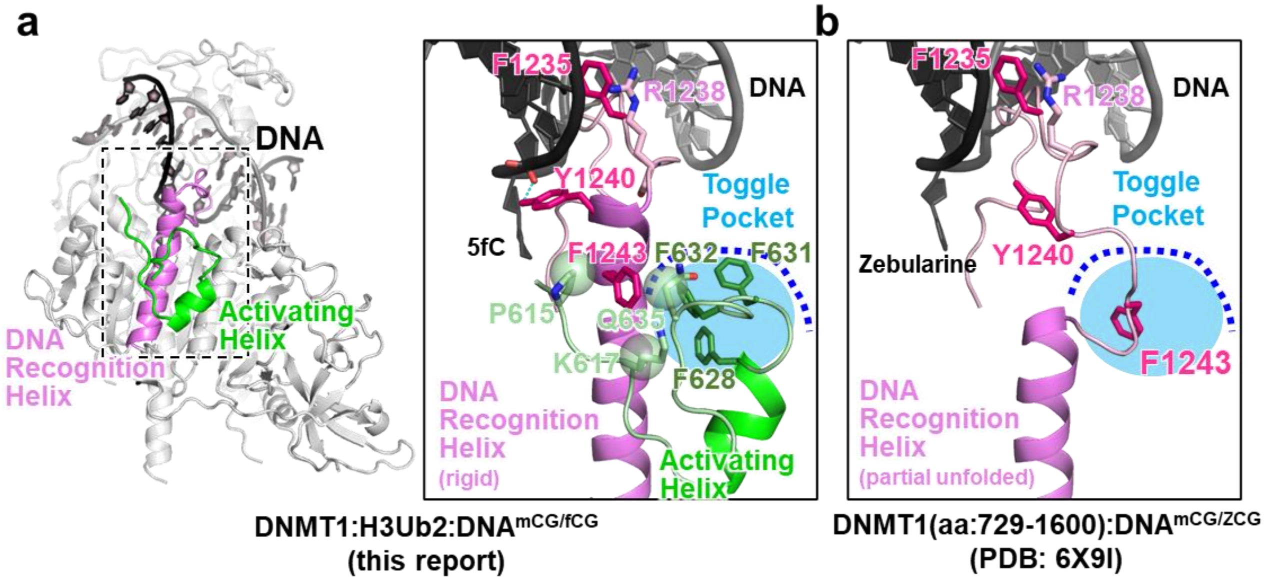

Supplementary Figure 9. Structural comparison of DNA recognition helix in the ternary complex and DNMT1:DNA$^{mCG/zCG}$ binary complex (aa: 729-1600, PDB:6X9I).

(a) Structure of DNA Recognition Helix (pink) and Activating Helix (green) of the ternary complex. Residues involving in recognition of DNA and interaction with the Toggle Pocket are shown as stick model. Toggle Pocket is highlighted as light-blue. The cognate DNA strands are colored as black and gray. **(b)** Structure of DNA Recognition Helix of DNMT1:DNA$^{mCG/zCG}$ binary complex (z: Zebularine). Color schemes are same as (a).

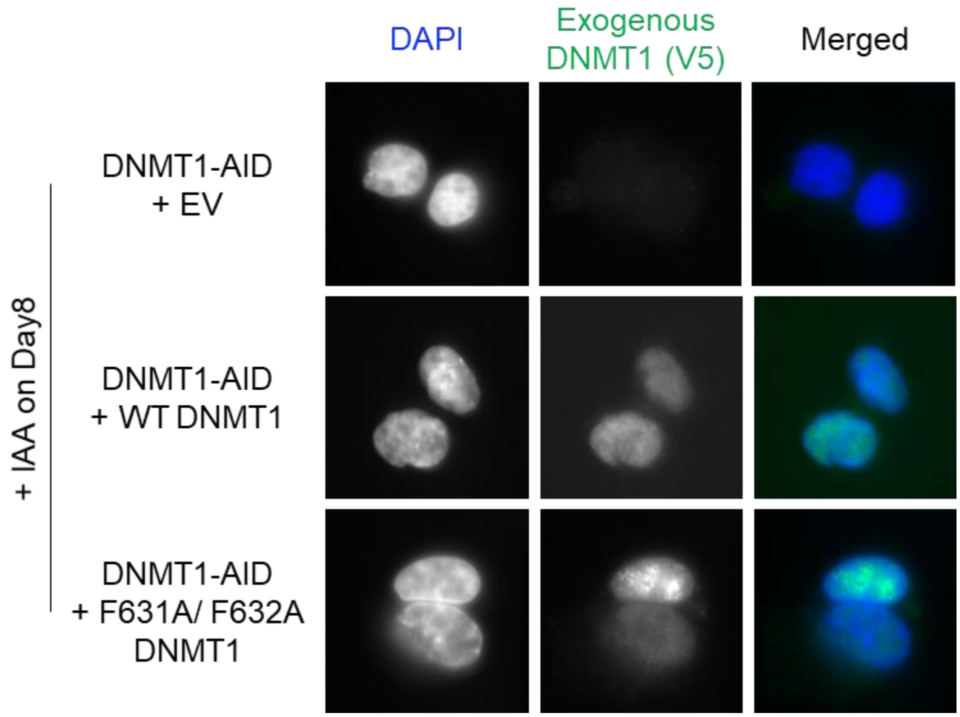

Supplementary Figure 10. Nuclear localization of Exogenous DNMT1.

The EV and exogenous V5 tagged DNMT1 (WT and F631A/F632A) were stably transfected to HCT116 cells expressing endogenous DNMT1-AID. The stable transfected cells were treated with IAA for 8 days. The nuclear was stained with DAPI, and exogenous DNMT1 was stained with V5 tag and second antibody. For merged images, the blue signal is corresponding to DAPI image, and the green signal is corresponding to exogenous DNMT1. Different images were captured at least five times.

a

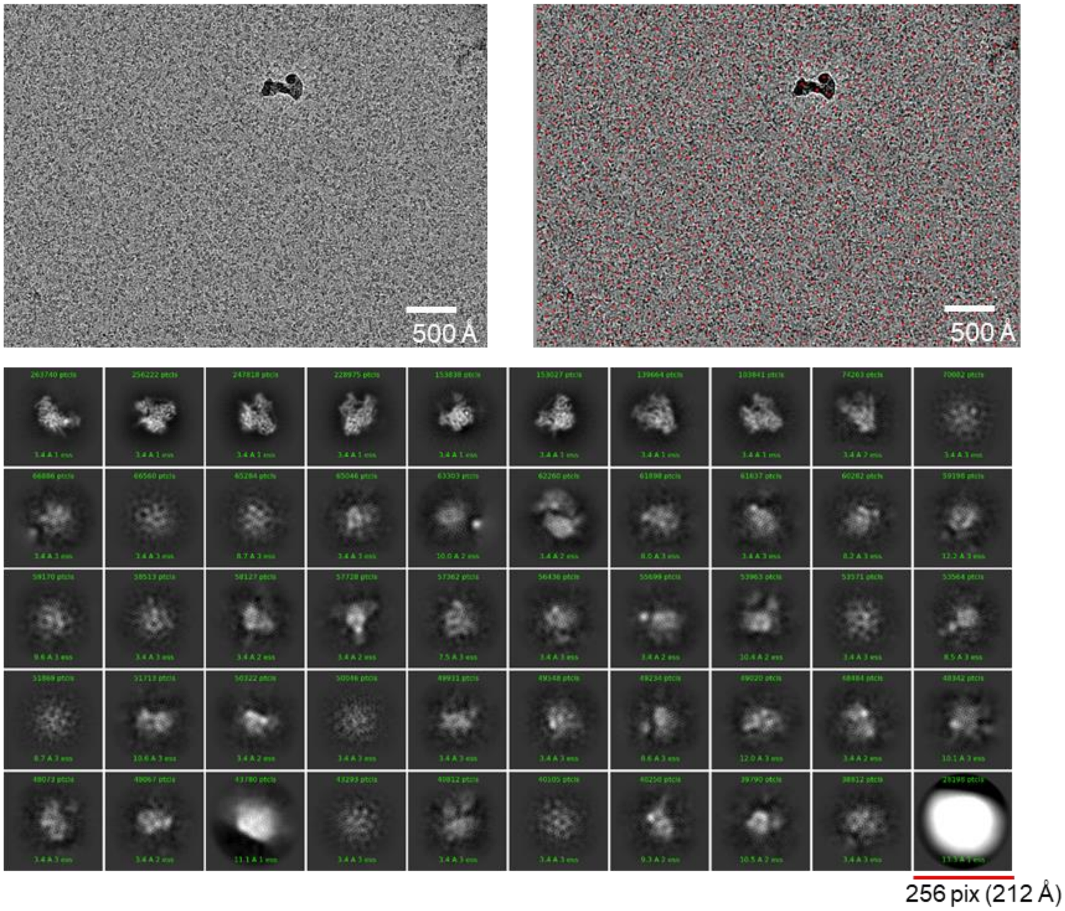

b

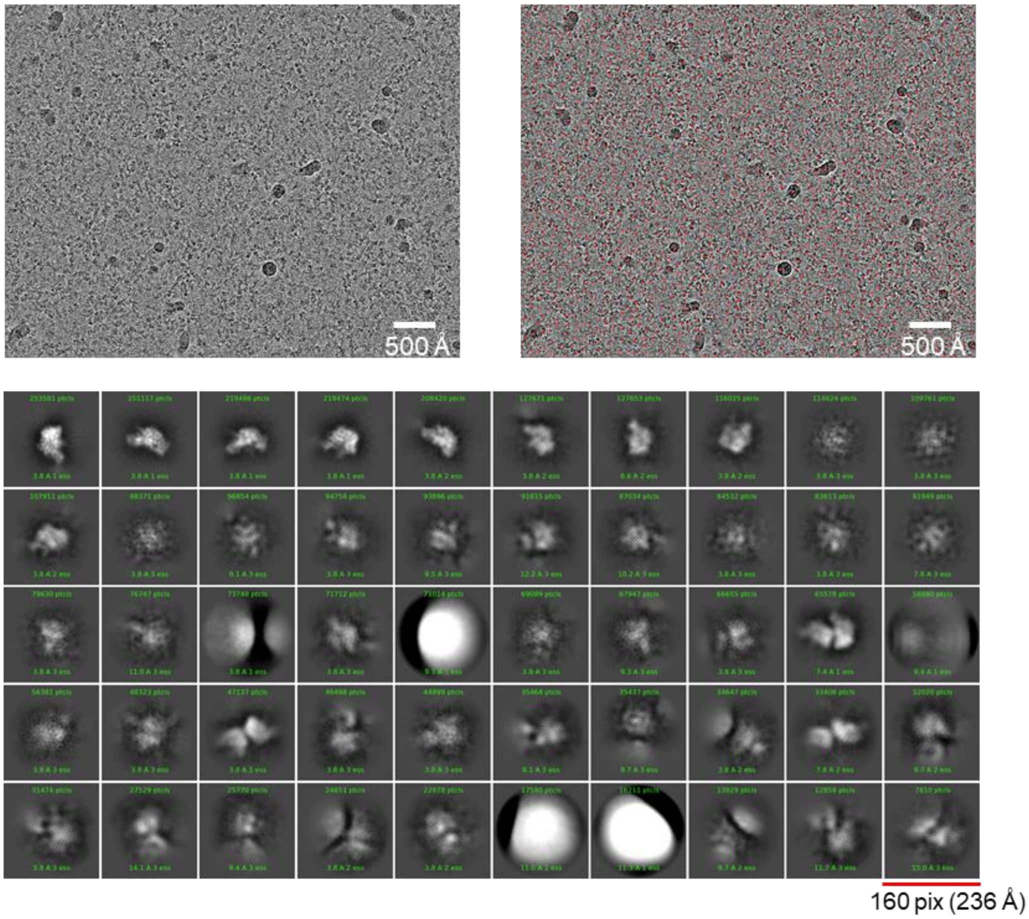

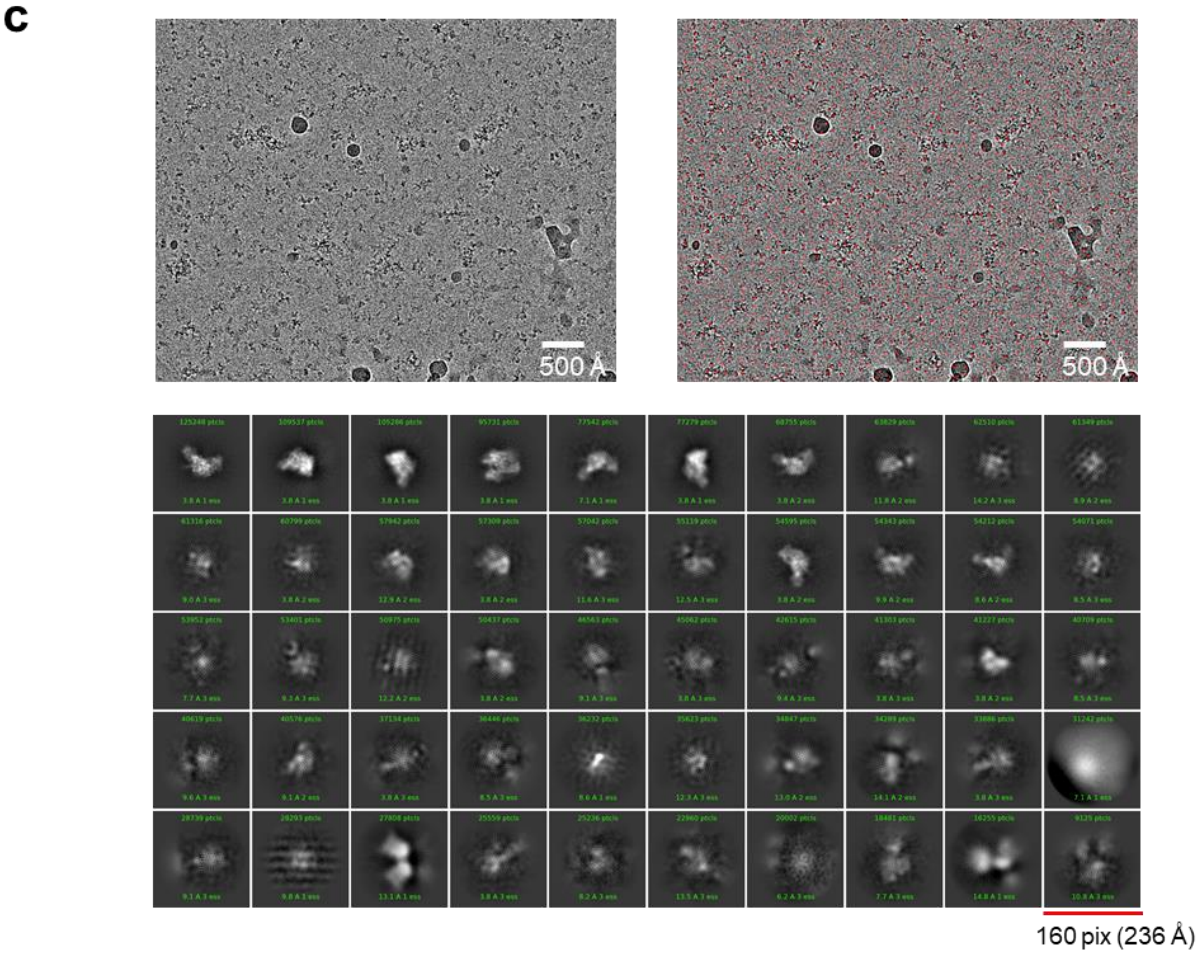

Supplementary Figure 11. Cryo-EM images of (a) DNMT1:H3Ub2:DNA$^{mCG/fCG}$ ternary complex, (b) apo-DNMT1 and (c) DNMT1:H3Ub2 binary complex.

Upper left panel displays motion corrected example of cryo-EM image and upper right shows initial picked particles marked with red dot in micrograph. Lower panel is initial reference-free 2D class averages.

Supplementary Tables

Supplementary Table 1. Cryo-EM data collection and refinement statistics for DNMT1:H3Ub2:DNA$^{mCG/fCG}$

	CXXC-ordered	CXXC-disordered	Source
EMDB number	EMD-33200	EMD-33201	
PDB number	7XI9	7XIB	
**Data-collection parameters**			
Microscope	Titan Krios		
Detector	K3 with BioQuantum energy filter		
Magnification	105,000 ×		
Voltage (kV)	300		
Electron expose/frame (e^-/Å^2)	1		
Defocus range (µm)	0.8-1.8		
Pixel size (Å)	0.83		
Number of images	4088		
Initial particle images	3,798,046		cryoSPARC
Final particle images	138,662	897,446	cryoSPARC
Map Resolution (Å) global	2.52	2.23	cryoSPARC
FSC threshold	0.143		
Symmetry imposed	C1		
**Refinement**			
Initial models used	CXXC-disordered	6X9I	PDB
Model resolution (Å)	2.5	2.2	Phenix
FSC threshold	0.143		
Model composition			
Non-hydrogen atoms	7620	7178	Phenix
Residues (Protein/Nucleotide)	870/24	815/24	Phenix
Ligands (SAM, Zn)	1, 4	1, 2	Phenix
Waters	232	196	Phenix
B factor			
Protein (min/max/mean)	23.61/100.73/45.86	17.33/92.01/40.26	Phenix
Nucleotides (min/max/mean)	33.43/78.83/52.07	28.24/74.35/44.81	Phenix
Ligands (min/max/mean)	31.21/103.17/39.18	25.67/75.17/29.45	Phenix
Waters (min/max/mean)	22.92/59.59/37.72	18.94/54.46/31.37	Phenix
r.m.s deviations			
Bond length (Å)	0.004	0.004	Phenix
Bond angles (°)	0.642	0.712	Phenix
**Validation**			
Clash score	7	7	wwPDB
Poor rotamer (%)	4.72	4.14	wwPDB
Ramachandran outliers (%)	0	0	Phenix
Ramachandran allowed (%)	3.98	3.87	Phenix
Ramachandran favored (%)	96.02	96.13	Phenix

Supplementary Table 2. Cryo-EM data collection and refinement statistics of apo-DNMT1 and DNMT1:H3Ub2 binary complex

	**apo-DNMT1**	**DNMT1:H3Ub2^{iso}**	**Source**
EMDB number	EMD-33299	EMD-33298	
**Data-collection parameters**			
Microscope	Tecnai Arctica		
Detector	K2		
Magnification	23,500 ×		
Voltage (kV)	200		
Electron expose/frame (e^-/Å^2)	1.25		
Defocus range (μm)	0.8-1.4		
Pixel size (Å)	1.477		
Number of images	2,071	1,869	
Initial particle images	3,984,637	2,463,410	cryoSPARC
Final particle images	380,989	645,368	cryoSPARC
Map Resolution (Å) global	3.45	3.52	cryoSPARC
FSC threshold	0.143		
Symmetry imposed	C1		

Supplementary Table 3. SAXS data collection and scattering derived parameters of apo-DNMT1 and DNMT1:H3Ub2^{S-S} binary complex.

a) Sample details

Sample name	**apo-DNMT1**	**DNMT1:H3Ub2^{S-S}**
Organism	*Homo sapiens sapiens*	
UniProt sequence ID	P26358 (DNMT1)	P26358 (DNMT1)<br>Q6NXT2 (Histone H3)<br>P0CG48 (Ubiquitin)
Extinction coefficient ε	161010	152530
Calculated monomeric Mr from sequence (kDa)	142.9 (DNMT1$_{351-1616}$)	163.9:<br>[142.9 (DNMT1$_{351-1616}$) + 17.2 (two ubiquitin) + 3.8 (H3$_{1-37W}$)]
HPLC ssystem	Nexera/Prominence-I (Shimazu)	
SEC Column	Superdex® 200 Increase 10/300 GL	
Temperature (K)	293	
Injection volume (µL)	100	
Loading concentration (mg/mL)	10	
Flow rate (mL/min)	0.05	
SEC buffer	20 mM Tris-HCl (pH 8.0), 150 mM NaCl and 5% glycerol	

b) Data-collection parameters

Beamline	Photon Factory BL-10C	
Detector	Pilatus3 2 M	
Sample-to-detector distance (mm)	2,082	
Wavelength (Å)	1.5	
q range (Å^{-1})	0.00605 - 0.264	0.00502 - 0.264
Exposure time (s)	20/frame	
Flux (photons/s)	1.1×10^{11}	
Beam size	0.63 mm (H) × 0.18 mm (V)	
Concentration range (mg/mL)	0.649 - 1.29	0.763 - 1.61
Absolute scaling method	Using the scattering intensity of water	
Normalization	To transmitted intensity by beam-stop counter	

c) Structural parameters

**Guinier analysis**		
$I(0)$ (cm^{-1})	0.1 ± 4.8 E^{-4}	0.11 ± 4.5 E^{-4}
R_g (Å)	39.0 ± 0.3 Å	43.7 ± 0.3 Å
q-range (Å^{-1}), point range	0.30-1.30, 11-158	0.31-1.30, 13-144
***P*(*r*) analysis**		
$I(0)$ (cm^{-1})	0.101 ± 4.75E^{-4}	0.112 ± 3.67 E^{-4}
R_g (Å)	39.6 ±0.3Å	46.5 ± 0.2 Å
D_{max} (Å)	139.3	155.4

q-range (Å^{-1}), point range	0.0078-0.2048, 11-1151	0.0071-0.1820, 13-1029
Porod volume estimate (Å^3)	247,275	276,939
Dry volume calculated from sequence (Å^3)	172,917	198,306
Partial specific volume (cm^3 g^{-1})	0.743	
Molecular mass Mr [from V_c] (kDa)	146.3	167.9
Calculated monomeric Mr from sequence (kDa)	142.9	163.9

d) Software employed

Primary data reduction	SAngler 2.1.39
Guinier Analysis	AutoGuinier (ATSAS 3.0.1)
Zero-concentration Extrapolation	SerialAnalyzer 1.3.1
Data processing	PRIMUS (ATSAS 3.0.1)

References

1. Ishiyama, S. *et al.* Structure of the Dnmt1 Reader Module Complexed with a Unique Two-Mono-Ubiquitin Mark on Histone H3 Reveals the Basis for DNA Methylation Maintenance. *Molecular Cell* **68**, 350-360.e7 (2017).
2. Zhang, Z. M. *et al.* Crystal Structure of Human DNA Methyltransferase 1. *Journal of molecular biology* **427**, 2520–2531 (2015).
3. Takeshita, K. *et al.* Structural insight into maintenance methylation by mouse DNA methyltransferase 1 (Dnmt1). *Proceedings of the National Academy of Sciences of the United States of America* **108**, 9055–9059 (2011).
4. Syeda, F. *et al.* The replication focus targeting sequence (RFTS) domain is a DNA-competitive inhibitor of Dnmt1. *The Journal of biological chemistry* **286**, 15344–15351 (2011).
5. Pappalardi, M. B. *et al.* Discovery of a first-in-class reversible DNMT1-selective inhibitor with improved tolerability and efficacy in acute myeloid leukemia. *Nature Cancer 2021 2:10* **2**, 1002–1017 (2021).
6. Adam, S. *et al.* DNA sequence-dependent activity and base flipping mechanisms of DNMT1 regulate genome-wide DNA methylation. *Nature Communications 2020 11:1* **11**, 1–15 (2020).
7. Song, J., Teplova, M., Ishibe-Murakami, S. & Patel, D. J. Structure-based mechanistic insights into DNMT1-mediated maintenance DNA methylation. *Science* **335**, 709–712 (2012).
